# National Kidney Month — March 2014

**Published:** 2014-03-07

March is designated National Kidney Month to raise awareness about the prevention and early detection of kidney disease. In 2011, kidney diseases were the ninth leading cause of death in the United States (1). More than 10% (>20 million) of U.S. adults aged ≥20 years have chronic kidney disease (CKD) (2). The chances of having CKD increase with age; the disease is most common among adults aged >70 years.

In collaboration with partner agencies and organizations, CDC released and continues to update the CKD Surveillance System website (http://nccd.cdc.gov/ckd) to document and monitor over time the number of cases of CKD and its risk factors in the United States. The website also provides the means for tracking progress toward achieving *Healthy People 2020* objectives to prevent, detect, and manage CKD (3), and for evaluating, monitoring, and implementing quality improvement efforts by federal and nonfederal agencies.

CDC and its partners developed and disseminated the *National Chronic Kidney Disease Fact Sheet, 2014*, a consensus document regarding CKD in the United States that includes data on prevalence by race/ethnicity, risk factors, and health consequences (2). Diabetes and high blood pressure are major risk factors for CKD, and controlling these two factors can prevent or delay CKD and improve health outcomes (2). Information about kidney disease prevention and control is available at http://www.nkdep.nih.gov. Information about CDC’s CKD Initiative is available at http://www.cdc.gov/ckd.
